# Can A Supplemental Hysterectomy Prevention Bundle Result In Reduction Of Surgical Site Infections?

**DOI:** 10.1017/ash.2025.406

**Published:** 2025-09-24

**Authors:** Aarikha D’Souza, Kisha McIntyre

**Affiliations:** 1 Banner Health; 2 Banner Health

## Abstract

**Background:** Surgical Site Infections (SSIs) are a major cause of morbidity resulting in devastating patient outcomes following an Abdominal Hysterectomy (HYST) procedure. No single intervention has demonstrated reduction in SSI rates, however, bundling prevention strategies have demonstrated reduction in SSI. In addition to our organization’s systemwide surgical site infection prevention bundle, we developed a supplemental bundle of focused strategies specific to abdominal hysterectomy procedures, to address a 37.26% increase in Abdominal Hysterectomy Standardized Infection Ratios (SIRs) in 2021. **Methods:** In 2021, a supplemental hysterectomy specific bundle was developed and implemented in three facilities within our health system that were experiencing increased HYST SIRs. After review of current literature, the following four strategies were included for the supplemental bundle for all abdominal hysterectomy procedures (open, laparoscopic, and robotic); the utilization of 500mg Metronidazole with Cefazolin as part of surgical antimicrobial prophylaxis, for cases where: anticipated bowel involvement occurs and for oncology patients with complex hysterectomies; the use of standardized vaginal and perineal preparation using either chlorhexidine (CHG) or Povidone Iodine (PVI); the use of a separate sterile closing tray; and changing of gown and gloves by surgical team, prior to going to abdomen from vaginal area. Compliance with the prevention strategies were measured during this period and SSI SIRs were reviewed monthly with overall trends monitored. The National Healthcare Safety Network (NHSN) criteria for SSI were used to assess for SSI after hysterectomy. **Results:** The SIR for HYST procedures in 2021 was 1.083 with 23 SSIs identified from 2339 abdominal hysterectomy procedures performed. Immediately following the implementation of the supplemental bundle at three facilities, the SIR decreased by 39% to 0.661 in 2022 with 11 SSIs identified from 1842 procedures performed. The HYST SIR outcomes were 0.782 in 2023 and currently at 0.979 through July 2024. Compliance during the intervention period ranged from 93.9% to 94.6%, and surgical antimicrobial prophylaxis compliance increased by 4% to 89.35% at these three facilities. **Conclusion:** Bundled interventions when employed, demonstrate benefit from the synergistic effects of multiple strategies decreasing the outcome rate of surgical site infections as compared to a single intervention. Establishing a standardized abdominal hysterectomy bundle, allows for minimal variation for patients undergoing abdominal hysterectomy procedures when adherence is at its maximum. Our goal is to expand systemwide based upon the successes from the three facilities, to achieve as close to zero postoperative infections by implementing evidence-based practices performed as a comprehensive bundle.

